# GATA3 positive spindle cell neoplasm involving the liver diagnosed as metastatic sarcomatoid chromophobe renal cell carcinoma at autopsy

**DOI:** 10.1111/his.15518

**Published:** 2025-07-21

**Authors:** Rangsinee Nusapan, Amer Abu Alfa, Agnes Balla, Sharon Mount, John M Kennedy

**Affiliations:** ^1^ Department of Pathology and Laboratory Medicine University of Vermont Medical Center Burlington Vermont USA; ^2^ Larner College of Medicine at the University of Vermont Burlington Vermont USA

**Abbreviations**
ChRCC: chromophobe renal cell carcinoma
CT: computed tomography
H&E: hematoxylin and eosin
IHC: immunohistochemistry

## Introduction

GATA3 immunoreactivity is commonly used to support urothelial or breast origin in metastatic tumours. Chromophobe renal cell carcinoma (ChRCC), a rarely encountered tumour in the metastatic setting, is GATA3 positive in up to 50% of cases including clinically aggressive tumours with sarcomatoid differentiation.^1^, ^2^, ^3^ Sarcomatoid differentiation is uncommon in ChRCC, reported in 0.8%–8.0% of cases, but is more likely to be seen in advanced disease.^1^ We present a case of a GATA3+ malignant spindle cell neoplasm diagnosed on a liver biopsy that was determined to be metastatic sarcomatoid ChRCC at autopsy. Morphologic and immunohistochemical findings are reviewed, and diagnostic challenges are discussed.

## Case Report

**Figure 1:**
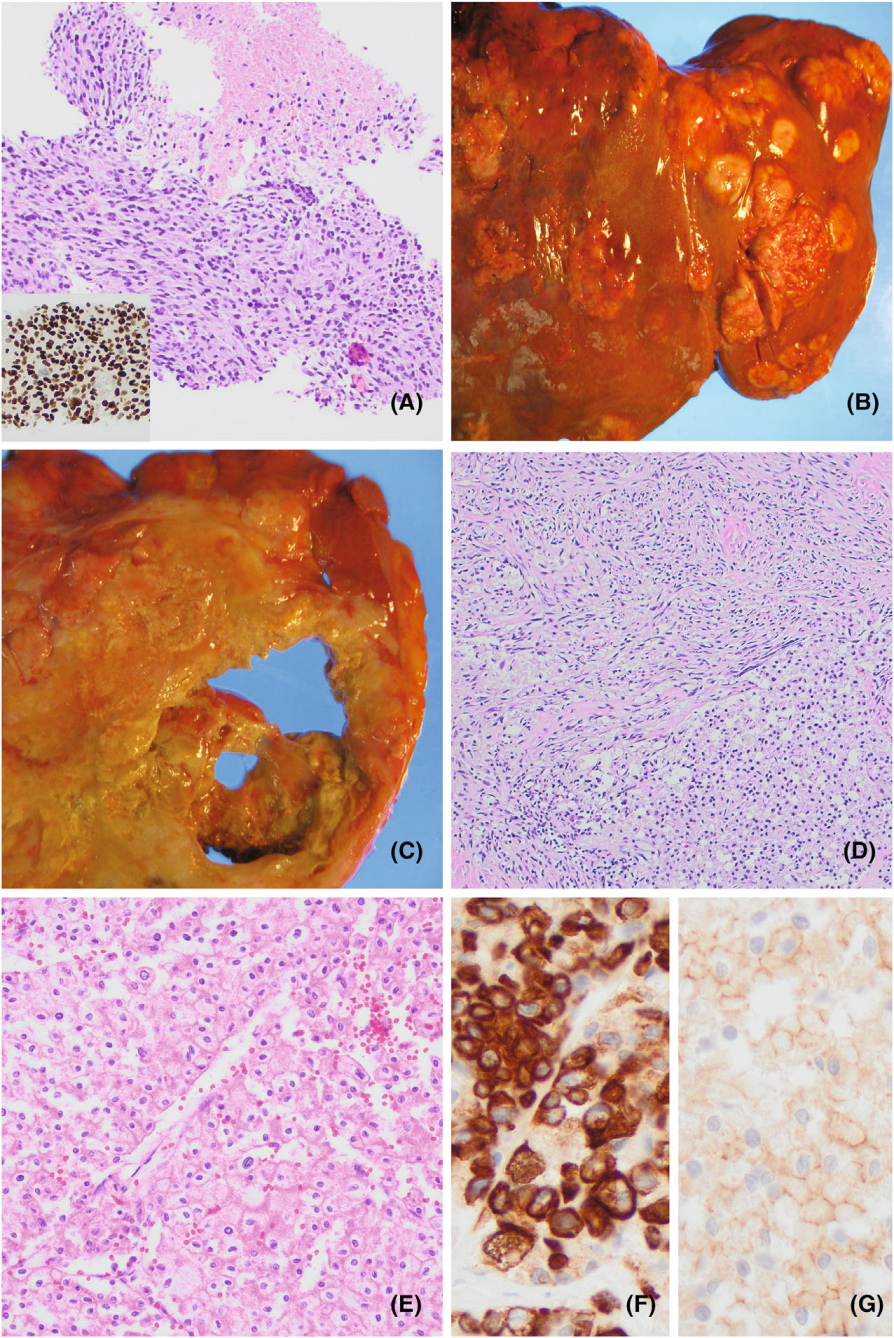
(**A**) Antemortem liver biopsy showing GATA3+ (inset) pleomorphic malignant spindle cells with tumour necrosis (H&E). (**B**) Liver at postmortem examination involved by multiple tumour nodules. (**C**) Cross section of kidney at postmortem examination, showing diffuse involvement by a necrotic tumour mass. (**D**) Microscopic postmortem examination showing the epithelioid ChRCC component (lower half) juxtaposed the sarcomatoid component (upper half) (H&E). (**E**) Microscopic postmortem examination showing solid growth of the epithelioid ChRCC component, with irregular nuclei, perinuclear halos and prominent cell membranes (H&E). (**F**) CK7 expression by IHC in epithelioid ChRCC component. (**G**) CD117 expression by IHC in epithelioid ChRCC component.

A 59‐year‐old female presented with abdominal pain. Abdominal computed tomography (CT) revealed a 16 cm left renal mass with intraperitoneal extension, multiple liver masses and mesenteric/omental lymphadenopathy. A liver biopsy showed a malignant pleomorphic spindle cell neoplasm with diffuse strong staining for GATA3, weak PAX8 expression and negativity for multiple cytokeratins (Figure 1A, Table 1). An epithelioid component was absent. A differential diagnosis of sarcomatoid carcinoma (renal versus urothelial primary) and high‐grade sarcoma was raised. The patient's metastatic cancer progressed rapidly and she died 3 weeks following her initial presentation.

**Table 1:** Immunophenotype of tumour in antemortem biopsy and postmortem autopsy

*Antemortem liver tumour biopsy immunohistochemical stains—pure sarcomatoid differentiation*	
Positive	EMA (weak), GATA3 (strong, diffuse), PAX8 (focal, weak), SMA (focal, weak), desmin (focal)
Negative	Keratin AE1/AE3, CAM 5.2, Keratin 34BE12, MNF116, CK7, CK20, P40, SOX10, DOG‐1, STAT6
*Postmortem kidney tumour immunohistochemical stains—Epithelioid component*	
Positive	Keratin AE1/AE3, PAX8 (moderate, patchy), CK7 (strong, diffuse), CD117, GATA3 (weak to moderate, patchy), FH (retained)
Negative	CA‐IX, TFE3 (non‐specific staining only), HMB45, Cathepsin‐K
*Postmortem kidney tumour immunohistochemical stains—Sarcomatoid component*	
Positive	Keratin AE1/AE3, PAX8 (weak, focal), GATA3 (weak), FH (retained)
Negative	CK7, CD117, CA‐IX, Cathepsin‐K, HMB45, TFE3

Postmortem macroscopic evaluation revealed extensive soft tumour nodules with necrosis involving the liver, left kidney and peritoneum (Figure 1B,C). The liver and kidney tumours showed predominately malignant spindle cells on postmortem microscopic examination, similar to the tumour seen on the antemortem liver biopsy. Focally, the kidney tumour showed solid nests of eosinophilic epithelioid tumour cells with features consistent with ChRCC, including irregular nuclear contours, perinuclear halos and prominent cell membranes (Figure 1D,E). The epithelioid component was positive for keratin AE1/AE3, PAX8, GATA3 (patchy), CK7 (Figure 1F) and CD117 (Figure 1G), also supporting the diagnosis of ChRCC. Additional immunohistochemical stains did not support rarer subtypes of renal cell carcinoma with eosinophilic cytoplasm (Table 1). A surface urothelial lesion in the renal pelvis was absent and morphologic features of urothelial carcinoma were not present. Overall, the postmortem findings supported the diagnosis of metastatic ChRCC with extensive sarcomatoid differentiation.

## Discussion

GATA3 is a sensitive marker commonly used to evaluate for carcinomas of urothelial and breast origin in the metastatic setting. However, GATA3 is non‐specific with variably reported positive expression rates in many neoplasms, including pancreatic adenocarcinoma, salivary gland neoplasms, malignant mesothelioma, squamous cell carcinomas of various sites, a subset of renal neoplasms such as ChRCC, choriocarcinoma and pheochromocytoma/paragangliomas.^2^ While reported at lower rates, GATA3 may be expressed in high‐grade sarcomas such as synovial sarcoma, leiomyosarcoma and malignant peripheral nerve sheath tumour. As our case report illustrates, considering the full morphologic and clinical context is essential to evaluating GATA3+ neoplasms in tissue biopsies at potential metastatic sites.

Our case demonstrated a malignant GATA3+ spindle cell neoplasm on antemortem liver biopsy, raising the differential diagnosis of sarcomatoid carcinoma and high‐grade sarcoma. There was no epithelioid component or cytokeratin expression on the biopsy material to support sarcomatoid carcinoma; however, a high index of suspicion of sarcomatoid carcinoma of urothelial or renal origin remained due to the presence of a concurrent kidney mass. Primary renal sarcomas are rare (reported in 0.39% of nephrectomies), which made metastatic sarcoma less likely but not excluded.^1^ The GATA3 expression suggested sarcomatoid urothelial carcinoma, although the awareness of GATA3 expression in a subset of renal cell carcinomas and high‐grade sarcomas led to including sarcomatoid renal cell carcinoma and sarcoma in the differential diagnosis. The observed weak PAX8 expression was of limited utility, as weak expression may be seen in upper tract urothelial carcinomas and may show attenuated to negative expression in a sarcomatoid renal cell carcinoma. A definitive diagnosis of sarcomatoid ChRCC was rendered only after extensive sampling of the kidney mass on postmortem examination revealed a focal epithelioid component of ChRCC. In high‐grade spindle cell neoplasms, the definitive aetiology of sarcomatoid carcinoma may not be identifiable on biopsy, resection specimen or postmortem examination without extensive sampling for the epithelioid component.

## Conclusion

GATA3 is expressed in a subset of renal cell carcinomas, particularly ChRCC with sarcomatoid differentiation. In the setting of a kidney mass with a biopsy at a metastatic site showing a GATA3 positive spindle cell neoplasm, the differential diagnosis should include sarcomatoid ChRCC.

## Author contributions

RN: writing—original draft. SM: conceptualization and supervision. JMK: conceptualization, supervision, writing – reviewing and editing. All authors contributed case material and/or interpreted surgical pathology/autopsy findings. All authors revised and approved the final manuscript. Authorship credit should be based only on (1) substantial contributions to conception and design or acquisition of data or analysis and interpretation of data; (2) drafting the article or revising it critically for important intellectual content; (3) final approval of the version to be published. Provision of funding, collection of data or general supervision of the research group alone does not justify authorship.

## Funding information

This research did not receive any specific grant from funding agencies in the public, commercial or not‐for‐profit sectors.

## Conflict of Interest

The authors declare that they have no known competing financial interests or personal relationships that could have appeared to influence the work reported in this paper.

## Patient consent statement

The patient's next of kin provided written consent for the publication of this report.

## Data Availability

Data sharing is not applicable to this article, as no data‐sets were generated or analysed during the current study.
